# Polymer-Based Mass Cytometry Reagents: Synthesis and Biomedical Applications

**DOI:** 10.3390/molecules30143034

**Published:** 2025-07-19

**Authors:** Yin-Feng Wang, Wenying Wu, Ya-Hui Ge

**Affiliations:** Institute of Advanced Clinical Medicine, Biomedical Engineering Department, Peking University, Beijing 100191, China

**Keywords:** mass cytometry, metal-chelating polymers, macrocyclic/acyclic chelators, polymeric particles, metal–organic frameworks

## Abstract

Mass cytometry has promoted the development of single-cell analysis by enabling the highly multiplexed detection of cellular markers using metal-tagged antibodies or cells. Polymer-based mass cytometry reagents have played a critical role in this technique due to their structural versatility, high metal-loading capacity, and sensitivity. This review comprehensively examines the advances in polymer-based reagents for mass cytometry, focusing on their design principles, synthetic strategies, and biomedical applications. We systematically analyze three key categories: metal-chelating polymers with macrocyclic/acyclic chelators developed through controlled polymerization techniques, polymeric particles including encoded microspheres and semiconducting polymer dots, and emerging metal–organic frameworks with high metal-loading capacities. The discussion highlights how these engineered materials overcome spectral limitations of conventional flow cytometry while addressing current challenges in sensitivity, and multiplexing capacity. Finally, we outline current challenges and future research directions for developing polymer probes in single-cell mass cytometry.

## 1. Introduction

Mass cytometry (CyTOF, time-of-flight cytometry), often recognized as “next-generation single-cell flow cytometry” [1], represents a groundbreaking advancement in cellular analysis by combining the high sensitivity and multiplex detection capabilities of inorganic mass spectrometry with single-cell technologies [2,3,4]. Unlike conventional fluorescence-based flow cytometry, which faces limitations due to spectral overlap and autofluorescence [5,6,7,8], mass cytometry utilizes metal-tagged antibodies detected via time-of-flight mass spectrometry (TOF-MS), enabling the simultaneous quantification of over 40 cellular parameters at single-cell resolution. This innovation has provided opportunities for high-throughput, multi-dimensional single-cell profiling in complex biological systems. In 2001, Tanner [9] and Zhang [10] independently proposed an inductively coupled plasma detection method for the element labeled enzyme-linked immunosorbent assay, laying the foundation for this technology. Tanner’s team developed the first mass cytometer [11,12], which integrated inductively coupled plasma time-of-flight mass spectrometry (ICP-TOF-MS) for single-cell analysis. A major turning point came in 2011 when Nolan’s group successfully applied mass cytometry to the study of the differentiation lineage of human bone marrow hematopoietic stem cells [13], demonstrating its transformative potential in immunology, cell biology, and clinical research [14,15,16].

**Figure 1:**
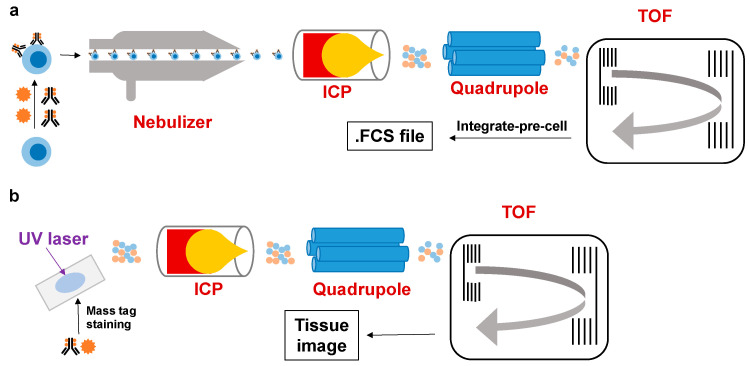
Depiction of (**a**) suspension mass cytometry and (**b**) imaging mass cytometry workflow.

CyTOF combines ICP-TOF-MS with conventional flow cytometry [17], with its workflow illustrated in Figure 1 [18,19]. Both suspension mass cytometry (SMC) and imaging mass cytometry (IMC) experiments require the staining of cells (for SMC) or tissue sections (for IMC) with reagents containing stable heavy metal isotopes. In SMC, stained cells in suspension are nebulized into single-cell droplets and introduced into the plasma torch of the ICP-TOF-MS instrument, where they undergo vaporization, atomization, and ionization. For IMC, stained tissue sections are placed in a slide chamber and then ablated by a UV laser in a raster pattern, generating an aerosolized cloud of tissue particles that enters the plasma torch. The resulting ion cloud then passes through a quadrupole filter into the TOF detector. Within a fixed flight distance, ions accelerated to the same kinetic energy are separated based on their mass-to-charge ratio (*m*/*z*). In SMC experiments, the isotopic reporter signals for each cell are quantified by integrating all scans per event. For IMC, the isotopic content measured by the TOF detector for each pixel ultimately forms a 2D image. Stable heavy metal isotope-conjugated reagents play critical roles throughout this process, making their development and optimization a key research focus in mass cytometry technology.

Over the past two decades, mass cytometry reagents have undergone significant advancements, with major categories including polymers, small molecules, and nanoparticles (NPs) [19,20,21]. Among these, polymer-based reagents stand out due to their unique structural properties, which allow multiple ligands to be conjugated onto a single molecule, thereby enabling higher metal-loading capacity, enhanced signal intensity, and greater modularity in probe design. Currently, metal-chelating polymers (MCPs) represent the most well-established class of mass cytometry reagents, with Maxpar^®^ reagents (Fluidigm, CA, USA) being a prime example widely used in conventional immunoassays. Beyond these, polymeric particles and metal–organic frameworks (MOFs)—a class of coordination polymers formed through the self-assembly of multidentate organic ligands containing oxygen or nitrogen and inorganic metal ions into supramolecular microporous networks [22,23,24,25]—have also emerged as promising platforms in recent years.

Given these developments, this review focuses on polymer-based mass cytometry reagents, covering traditional MCPs with macrocyclic/acyclic chelators, polymeric particles (e.g., polystyrene (PS)-based carriers), and MOF-based mass tag reagents. We discuss their synthetic strategies, structural design principles, and key achievements in mass cytometry applications, providing insights into how these advanced materials are pushing the boundaries of high-dimensional single-cell analysis.

## 2. Metal-Chelating Polymers as Mass Cytometry Reagents

**Figure 2:**
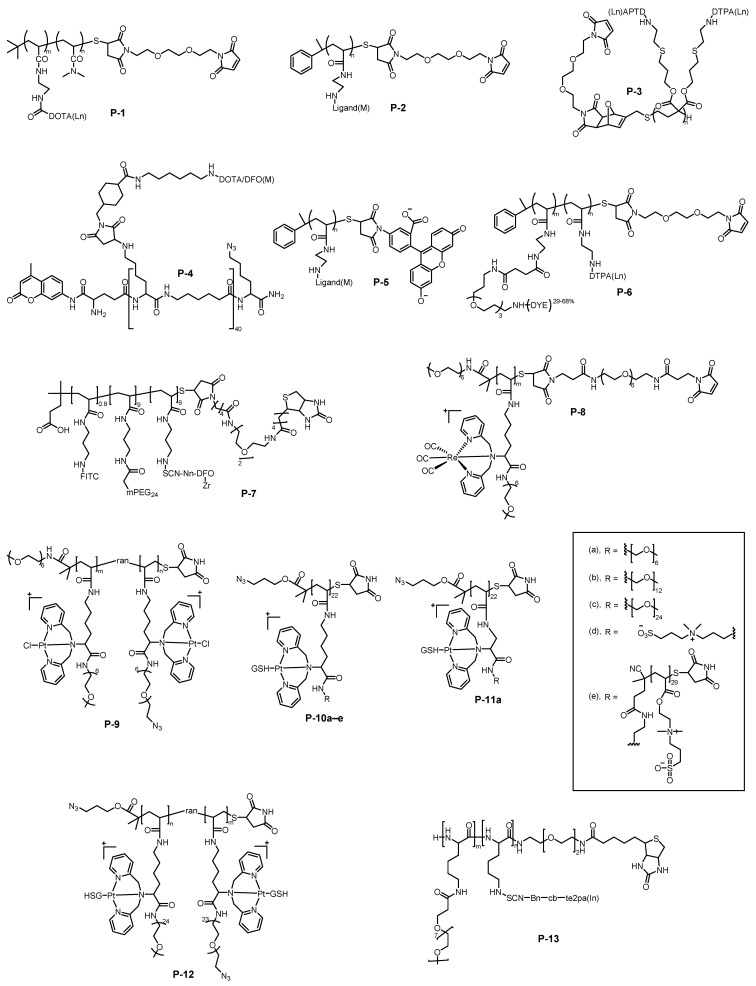
Chemical structures of various MCPs that have been employed as mass cytometry reagents.

The development of MCPs has rapidly advanced the progress of mass cytometry by enabling highly multiplexed single-cell analysis. In their pioneering 2007 study [26], Winnik and colleagues employed controlled reversible addition–fragmentation chain transfer (RAFT) polymerization to synthesize a well-defined MCP (**P-1**, Figure 2) with an average degree of polymerization of 52 and narrow dispersity (PDI = 1.15). This architectural precision enabled each polymer chain to carry approximately 33 DOTA (1,4,7,10-tetraazacyclododecane-1,4,7,10-tetraacetic acid) chelators, creating a high payload capacity for lanthanide (Ln) ions. When conjugated to antibodies through maleimide-thiol chemistry, these polymer tags demonstrated remarkable performance, enhancing the ICP-MS detection sensitivity by 100-200-fold compared to nonspecific binding (NSB) signals. They achieved the simultaneous detection of five leukemia biomarkers (CD33-Pr, CD34-Tb, CD38-Ho, CD45-Eu, and CD54-Tm) in myeloid and monocytic cell lines. Remarkably, their method could quantitatively analyze two protein markers differing by 500-fold in expression levels within a single assay. This breakthrough overcame the spectral overlap limitations of fluorescent labeling and established a new paradigm for multiparameter single-cell analysis, laying the foundation for the subsequent commercialization of Maxpar^®^ reagents.

**Figure 3:**
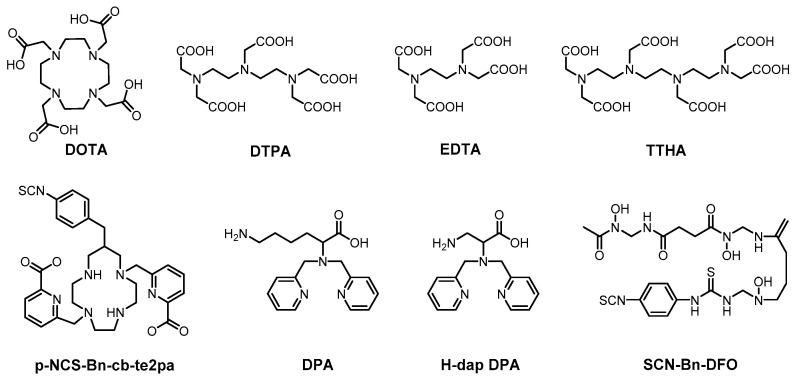
Chemical structures of macrocyclic/acyclic chelators that have been employed as MCP mass cytometry reagents.

Currently, the majority of commercially available mass cytometry reagents belong to the category of MCPs, owing to their unique combination of structural versatility and metal-coordination capacity. These polymers typically incorporate high densities of specialized chelating ligands designed for optimal metal binding. Lanthanides remain the elements of choice for several compelling reasons: (1) their chemically homogeneous +3 oxidation state ensures consistent coordination behavior across the series [27,28,29]; (2) their low natural abundance in cells minimizes background interference [12,30]; and (3) their hard Lewis acid characteristic favors stable complexes with oxygen/nitrogen-donor ligands [31]. Among various chelators, the macrocyclic DOTA stands out for forming stable lanthanide complexes with remarkable kinetic inertness and thermodynamic stability [31,32,33]. The linear DTPA (diethylenetriaminepentaacetic acid) chelator is another popular choice for lanthanide coordination [34], which can quickly bind metal ions and be characterized by isothermal titration calorimetry (ITC). However, both DOTA and DTPA show limitations when developing reagents for other metal isotopes beyond lanthanides. To expand the metal isotope repertoire of MCPs and fully exploit the capability of mass cytometry to resolve >100 parameters, researchers have introduced novel metal-chelating macrocyclic/acyclic ligands (Figure 3), including TEPA (tetraethylenepentamine) derivatives, EDTA (ethylenediaminetetraacetic acid), TTHA (triethylenetetramine-*N*,*N*,*N*′,*N*′′,*N*′′′,*N*′′′-hexaacetic acid), DPA (2,2′-dipicolylamine), and DFO (deferoxamine).

This section focuses on the synthesis methods of MCPs and their classification based on coordinated metals: transition metal-chelating polymers, and main-group metal-chelating polymers.

### 2.1. Synthesis of MCPs

**Figure 4:**
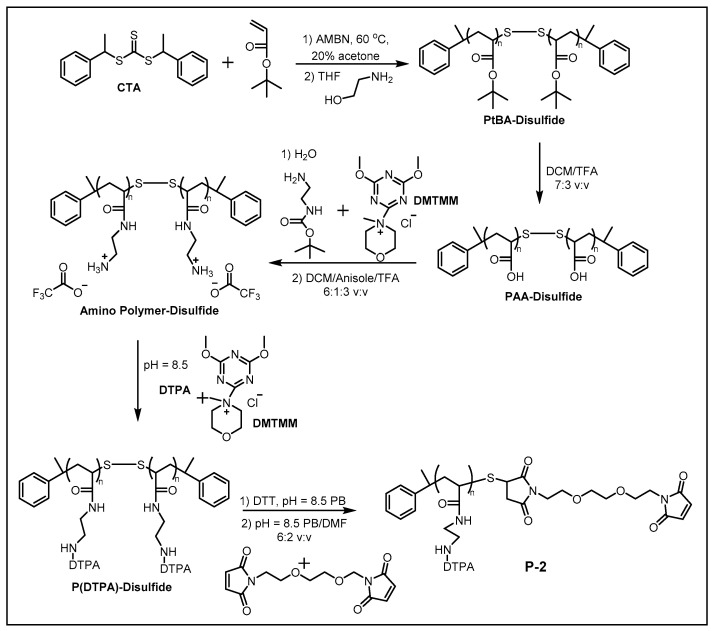
Synthesis of polymer **P-2** via reversible addition–fragmentation chain transfer polymerization [35].

At present, the synthesis of MCPs primarily relies on RAFT polymerization, which is a controlled free radical polymerization technique. To illustrate this methodology, we detail the step-by-step synthesis of polymer **P-2** as a representative example (Figure 4) [35]. The synthesis of **P-2** began with the RAFT polymerization of *t*-butyl acrylate using di-1-phenylethyl trithiocarbonate (CTA) as a chain transfer agent, yielding poly(*t*-butyl acrylate) (PtBA) with controlled degrees of polymerization (*n* ≈ 67 or 79) and narrow dispersity (PDI ≤ 1.17). The trithiocarbonate end group was aminolyzed with aminoethanol and oxidized to a disulfide-linked dimer, protecting the thiol termini during subsequent modifications. The *t*-butyl esters were then deprotected with trifluoroacetic acid (TFA) to generate poly(acrylic acid) (PAA), followed by coupling with *t*-BOC-ethylenediamine using DMTMM (4-(4,6-dimethoxy-1,3,5-triazin-2-yl)-4-methylmorpholinium chloride) and subsequent BOC deprotection to introduce primary amines. These pendant amines were functionalized with DTPA via amide coupling, achieving near-quantitative ligand incorporation. The disulfide bridge was reduced with dithiothreitol (DTT), and the resulting thiols were reacted with a bismaleimide linker to introduce maleimide end groups (92% efficiency) for antibody conjugation. This modular approach enabled control over polymer length, ligand density, and bio-orthogonal functionality, making the MCPs ideal for multiplexed mass cytometry applications.

**Figure 5:**
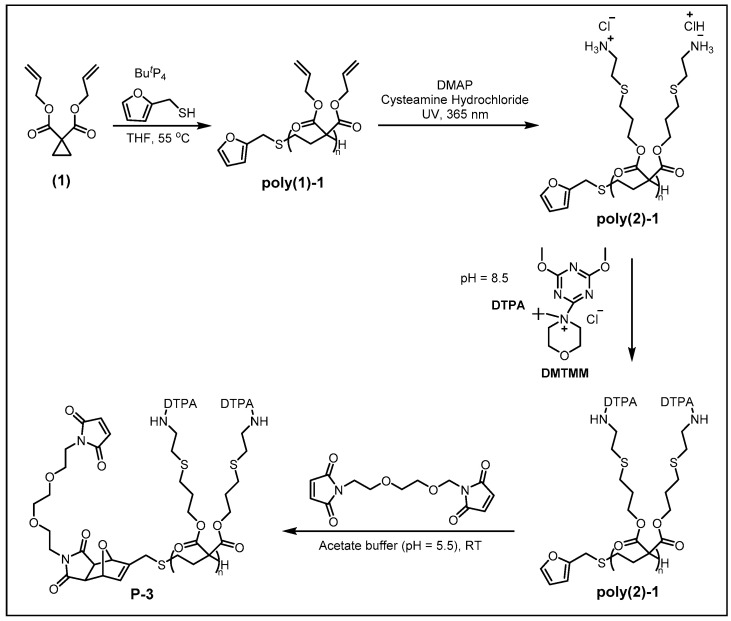
Synthesis of polymer **P-3** via anionic ring-opening polymerization [36].

Structurally, conventional (meth)acrylate-based polymers feature one substituent per two carbon atoms along the backbone. By contrast, polymers synthesized via the anionic ring-opening polymerization (ROP) of activated cyclopropanes exhibit a unique architecture with two geminal substituents on every third carbon atom. As shown in Figure 5, the synthesis of polymer **P-3** was achieved through the anionic ROP of diallyl cyclopropane-1,1-dicarboxylate, initiated by a 2-furanmethanethiol/phosphazene base (*t*-BuP_4_) system, which provided control over molecular weight and narrow dispersity (PDI ≤ 1.09) [36]. The resulting polymer featured furyl end-groups for subsequent bioconjugation and allyl pendant groups for ligand functionalization. A photoinitiated thiol-ene addition introduced primary amines onto the pendant groups, followed by coupling with DTPA using DMTMM to achieve high-density metal-chelating sites. The terminal furan was further functionalized via a Diels–Alder reaction with a bismaleimide linker, enabling covalent attachment to antibodies through Michael addition to thiols generated by disulfide reduction. This modular approach combined the advantages of anionic ROP (controlled architecture) and post-polymerization modifications (high functionality), yielding MCPs with high lanthanide-binding capacity (49.5 ± 6 Gd^3+^ ions per chain). However, during the pendant group modification, the coupling efficiency of DTPA to the side-chain amines was not quite quantitative, achieving only 75–90% of the theoretical loading when quantified by end-group analysis.

While RAFT and ROP techniques provide moderate control over polymer chain length, their inherent chain-growth mechanism lacks stepwise monomer addition, resulting in products with inevitable length dispersity (PDI > 1.0). Furthermore, the conventional approach of first incorporating chelating agents (e.g., DOTA, DTPA) into the polymer backbone followed by post-synthetic metalation faces fundamental limitations—the variable affinity constants of heavy metals for chelators preclude quantitative metal incorporation. By contrast, solid-phase peptide synthesis (SPPS) demonstrates the potential for absolute structural control by controlling monomer addition through means of protective groups, achieving near-perfect sequence fidelity [37,38,39,40]. The expanding repertoire of functionalized building blocks with precisely positioned reactive side chains now enables the rational design of polymeric scaffolds with defined chain lengths, structures, tailored hydrophobicity profiles, and spatially controlled functional group orientation, opening new possibilities for next-generation metal-chelating polymer development.

**Figure 6:**
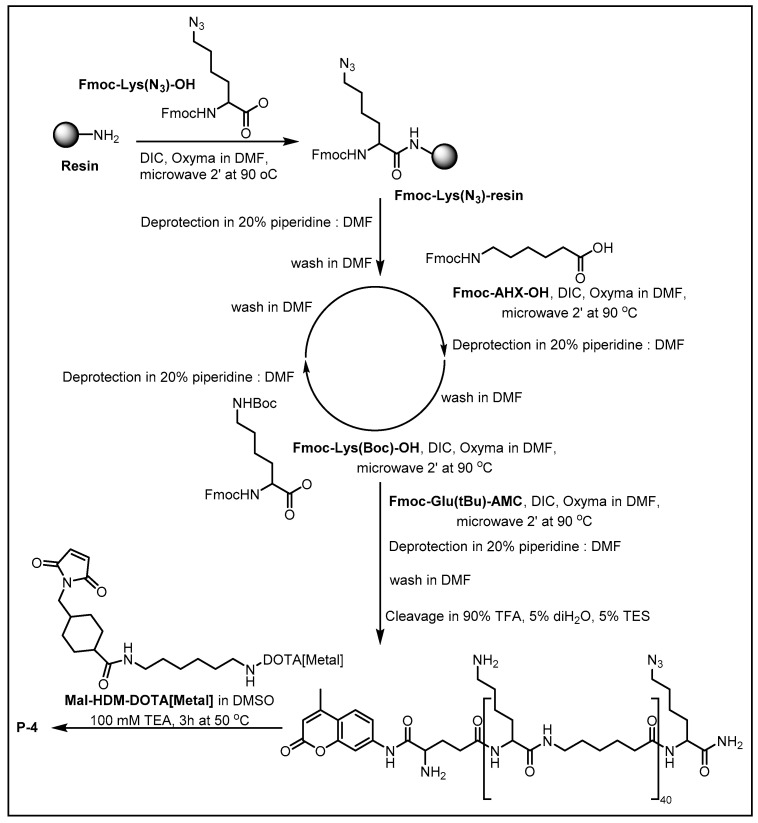
Synthesis of polymer **P-4** via solid-phase peptide synthesis [41].

Polymer **P-4** was selected as a representative example to describe the microwave-assisted SPPS for MCPs preparation (Figure 6) [41]. The synthesis employs DIC/Oxyma as coupling reagents for amide bond formation between amino and carboxyl groups. Prior to each monomer addition, the resin-bound intermediate undergoes Fmoc deprotection and thorough washing. The process begins by anchoring Fmoc-Lys(N_3_)-OH to the resin, followed by iterative cycles of coupling Fmoc-Lys(Boc)-OH and 6-aminohexanoic acid (Fmoc-AHX-OH) to extend the polymer backbone until the desired length is achieved. The chain is then capped with a fluorogenic tracer (7-amino-4-methylcoumarin, AMC). Subsequently, the polymer is cleaved from the resin using a TFA-based cleavage cocktail. Pre-synthesized metal–chelator complexes are conjugated to the polymer via maleimide-amine coupling on the lysine side chains, yielding the final MCP. While this method enables precise control over polymer length and metal loading, the incorporation of metal–chelator complexes is not quantitative, resulting in partial functionalization of the scaffold.

### 2.2. Transition Metal-Chelating Polymers

**Figure 7:**
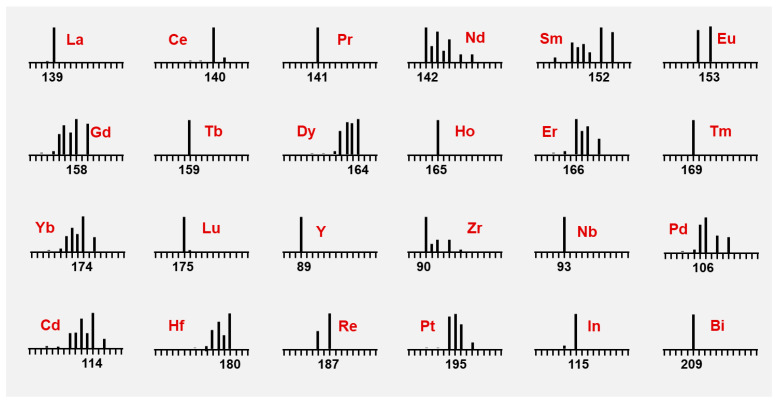
Isotopic natural abundances of metal elements used in MCPs [42].

Among transition metals, Ln isotopes are the most widely used in MCPs, with their natural abundance depicted in Figure 7 and Table 1. The Ln series, comprising 15 metallic elements from lanthanum (La, atomic number 57) to lutetium (Lu, 71), possesses a distinctive electronic configuration of [Xe]4f^0−14^ 5d^0−1^ 6s^2^. These elements predominantly exhibit a stable +3 oxidation state due to the relatively low ionization energy required to remove two 6s electrons and either one 5d or 4f electron. This characteristic, combined with their strong affinity for oxygen/nitrogen-containing ligands (e.g., macrocyclic DOTA, acyclic DTPA), enables the formation of exceptionally stable chelate complexes. These fundamental properties have established Ln-chelating polymers as indispensable reagents in mass cytometry, where they serve as the foundation for highly multiplexed single-cell analysis. This section systematically examines the change, design principles, and cutting-edge applications of lanthanide-chelating polymers that are pushing the development of high-dimensional cellular analysis.

Following the successful development of polymer **P-1** by Winnik, Nitz, and colleagues [26], further structural optimizations were made to the polymer. The modified synthesis protocol omitted detailed elaboration of the free thiol group and replaced the DOTA ligand with DTPA for Ln coordination [43]. The lectins were first modified on surface lysine residues using sulfosuccinimidyl-4-(*N*-maleimidomethyl)cyclohexane-1-carboxylate, enabling conjugation to thiol-containing polymers preloaded with Ln ions. This polymer design ensured stable metal loading while preserving lectin specificity. At the concentrations used in the assay, no interference between conjugates or Ln exchange between polymers was observed. By labeling different lectins with distinct Ln elements, they successfully achieved multiplexed detection, allowing for a simultaneous glycoprotein analysis. This work highlights the potential of polymer design in enhancing the sensitivity and multiplexing capacity of ICP-MS-based assays, paving the way for broader applications in glycobiology. In subsequent work, they expanded the application of Ln-tagged lectins. They synthesized a novel membrane stain, didodecyl-DTPA (DDD-Eu), which incorporates lanthanides into a lipid-like structure for stable bacterial labeling [44]. When combined with lectin-polymer conjugates (e.g., ConA-Tb and WGA-Ho), this method enabled the discrimination of Escherichia coli strains based on surface polysaccharides. However, CyTOF analysis of bacterial cells revealed that the signal intensity of each bacterial strain was lower than ideal. For enhanced multiplexing capabilities, employing element tags with higher atomic payloads would markedly boost detection sensitivity. Expanding the library of element-tagged probes will open new opportunities to study biological questions that were previously inaccessible to bulk assays.

The conventional thiol-maleimide chemistry used for labeling antibodies with maleimide-functionalized MCPs requires partial reduction of the antibody to generate reactive thiol groups. However, this approach has inherent limitations, particularly for labeling certain antibody classes (e.g., IgM) and biomolecules lacking cysteine residues. To overcome these constraints, Allo et al. [45] developed a novel class of MCPs with bio-orthogonal end groups. They synthesized a thiol-terminated **P-1** polymer precursor (prior to bismaleimide linker conjugation) and reacted it with transcyclooctene (TCO)–PEG3–maleimide to yield TCO-terminated MCPs. Similarly, azide-terminated MCPs were prepared by reacting the Fluidigm polymer with azido-PEG3–maleimide. These modified MCPs enable copper(I)-free strain-promoted alkyne-azide cycloaddition (SPAAC) or tetrazine-alkene click chemistry, where biomolecules containing -NH_2_ groups are selectively activated with dibenzocyclooctyne (DBCO) or tetrazine moieties, respectively. This strategy allows for the efficient and specific labeling of a broad range of biomolecules, including IgGs, IgMs, lectins, and small peptides, producing highly sensitive and selective reagents for mass cytometry and IMC. The study demonstrated the successful application of these MCPs in immunophenotyping, glycomics, and the development of dual-labeled (fluorescence and metal-tagged) reagents. In some cases, the click-chemistry-derived conjugates exhibited superior sensitivity and specificity compared to those generated via traditional thiol-maleimide conjugation. The integration of click chemistry with these advanced MCPs holds significant potential for developing a diverse array of high-sensitivity metal-containing reagents for proteomics and glycomics applications.

In 2010, Winnik and colleagues first synthesized polymer **P-2** and characterized its metal-binding capacity using ITC, demonstrating that each polymer chain (DPₙ = 79) binds 68 ± 7 Gd^3+^ ions [35]. When **P-2** was loaded with ^159^Tb and conjugated to secondary goat anti-mouse IgG, ICP-MS analysis revealed approximately 2.4 polymer chains per antibody, corresponding to an average of 161 ± 4 ^159^Tb atoms per antibody. These MCPs with different Ln isotopes (^141^Pr, ^142^Nd, ^144^Nd, ^146^Nd, ^152^Sm, ^156^Gd, ^158^Gd, ^159^Tb, ^162^Dy, ^165^Ho, ^170^Er) were then employed to label 11 distinct monoclonal primary antibodies, enabling multiplexed single-cell analysis of human umbilical cord blood via mass cytometry. The 11-parameter panel successfully resolved major immune cell populations, including lymphocytes, granulocytes, monocytes, and subsets of CD3^+^ T cells and B cells, without spectral overlap, highlighting the polymer’s utility in high-dimensional immunophenotyping.

In subsequent work, the team modified the **P-2** scaffold by replacing the bismaleimide linker at the terminus of the polymer chain with a fluorescein molecule. This modification, along with the incorporation of four distinct pendant polyaminocarboxylate ligands (EDTA, DTPA, TTHA, DOTA), resulted in the synthesis of the new polymer structure **P-5** [46]. This fluorescein tag enabled the rapid quantification of lanthanide, palladium, and platinum ion loading in the respective ligand-bearing polymers through combined UV/vis and ICP-MS measurements. The study demonstrated these polymers’ capacity for substantial metal incorporation. Based on these findings, the team prepared palladium (Pd)- and platinum (Pt)-loaded EDTA-containing polymers (**P-2**) for model mass cytometric immunoassays. While Ln-loaded polymers performed as anticipated, the Pd- and Pt-loaded MCPs exhibited unexpected behavior: they lost their antigen-binding capability but effectively stained dead cells, likely due to the soft metal ions forming mixed complexes with ligands encountered on the inside and/or outside of dead cells. The structural design of probes, particularly the selection of chelating ligands and the critical balance between charge and solubility, proved pivotal for this dual functionality. These results not only highlight challenges in designing MCPs for multiplexed detection but also reveal their novel application as robust dead-cell stains. Pd isotopes are commonly employed as cell barcoding. In one study, a combinatorial labeling system using three Pd tags with seven cadmium (Cd) tags demonstrated superior signal intensity without spectral interference with Ln detection channels [47].

The **P-2** scaffold was also modified by incorporating fluorescent dyes (FITC and DyLight dyes 405/549/649) into the side chains, yielding four dual-purpose MCPs (chemical structure **P-6**) [48]. These polymers retained ~70 DTPA chelating groups while carrying 2–6 fluorophores per chain and were conjugated to secondary antibodies via terminal maleimide linkers for staining cells in a four-plex assay. While CD3-FITC and CD45-DyLight 649 conjugates performed well in biological assays, CD13-DyLight 405 and CD38-DyLight 549 exhibited weak signals and NSB, underscoring challenges in dye compatibility and polymer antibody interactions. This work emphasized the potential of dual-purpose labeling for cross-validating mass cytometry and enabling fluorescence-based cytometry but also revealed limitations in dye selection and synthetic efficiency, particularly in maleimide functionalization.

**Figure 8:**
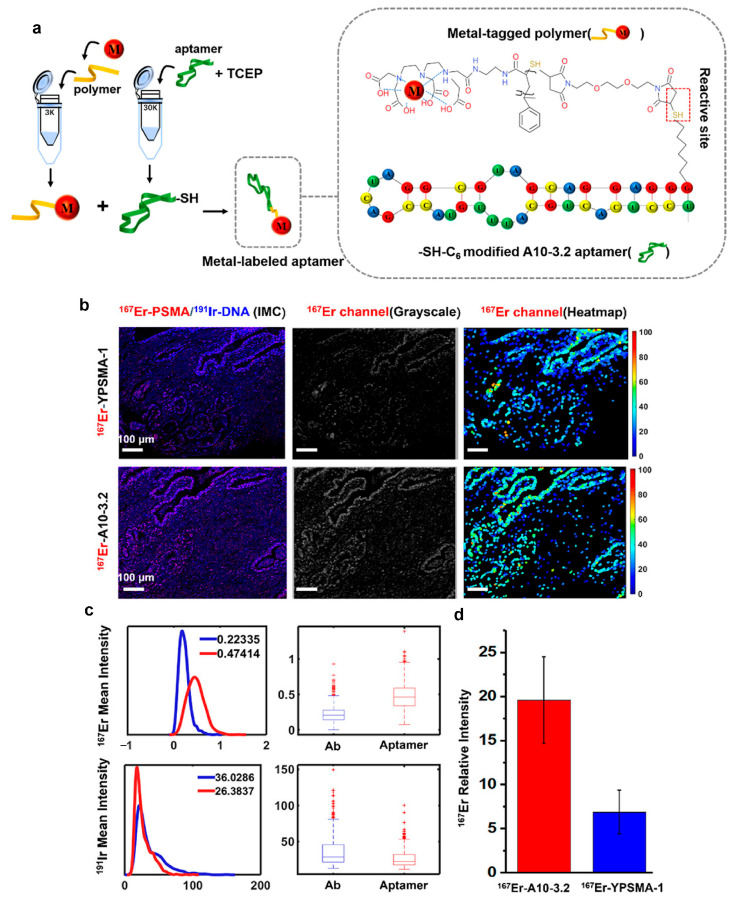
(**a**) Chemical synthesis of metal-labeled aptamer: metal-tagged polymer was conjugated with mercapto-modified A10-3.2 aptamer via maleimide and the thiol reaction. (**b**) IMC images of PaC tissue section probed by ^167^Er-YPSMA-1 and ^167^Er-A10-3.2 probes and corresponding grayscale images and heatmap images of the 167Er channel. Epitope retrieval temperature, 37 °C; scale bar, 100 μm. These pairs of images are equally scaled to allow for a direct visual comparison. (**c**) The histogram and box-plot for quantifying mean intensity of ^167^Er and ^191^Ir (red for ^167^Er-A10-3.2, blue for ^167^Er-YPSMA-1). (**d**) Quantification of the average ^167^Er relative intensity by taking the mean value per 100 cells (standard deviation is calculated based on random selection of 100 cells from the total cells). Reproduced with permission [49]. Copyright 2020, American Chemical Society.

Ding et al. [49] developed a structurally optimized metal-labeled aptamer nanoprobe (MAP), designated as ^167^Er-A10-3.2, by conjugating an ^167^Er-chelated **P-2** polymer with a truncated 37-nucleotide PSMA-specific RNA aptamer (A10-3.2) through maleimide-thiol linkage (Figure 8a). This metal-labeled aptamer probe specifically targets PSMA overexpression in epithelial cells while demonstrating no reactivity with background cells or cross-reactivity with non-PSMA-expressing tissues. The compact architecture (~5 nm in size, compared to ~20 nm for the antibody probe ^167^Er-YPSMA-1) capitalizes on the aptamer’s small size and high-affinity binding, combined with a polymeric scaffold containing 20-25 DTPA chelators to amplify metal signals. This design achieved a 3-fold higher sensitivity than antibody probes under low-temperature epitope retrieval conditions (37 °C) in prostate adenocarcinoma tissues (Figure 8b–d), enabling high-density labeling with minimal steric hindrance. While its advantages include multiplex compatibility (demonstrated through the simultaneous staining of dual metal labeled aptamers, ^167^Er-A10-3.2 and ^176^Yb-ΔPsap4#5), challenges remain, such as the need for broader validation across diverse biological systems.

In the development of MCPs, Winnik’s group [36] pioneered the synthesis of three well-defined MCPs (**P-3**) via anionic ROP. This approach represents a notable departure from their previous RAFT-polymerized counterparts. These polymers feature a unique geminal substitution pattern along the backbone, which optimizes chelator packing density while minimizing steric hindrance. **P-3** (DPn = 35, binding 49.5 ± 6 Ln^3+^ per chain) was successfully loaded with 10 distinct Ln isotopes to tag corresponding antibodies (CD3-^152^Sm, CD20-^171^Yb, CD45-^159^Tb, CD4-^142^Nd, CD8a-^146^Nd, CD38-^165^Ho, CD34-^169^Tm, CD14-^176^Yb, CD33-^164^Dy, CD15-^170^Er). When applied to 10-plex single-cell analysis of human peripheral blood mononuclear cells (PBMCs) by mass cytometry, this system successfully quantified the antibody-binding capacity for 10 distinct cell surface antigens. Notably, the CD38-^165^Ho- and CD34-^169^Tm-tagged antibodies demonstrated high sensitivity in identifying a very small fraction (0.05%) of hematopoietic stem cells. While this work highlights the critical role of polymer architecture in advancing diagnostic probes, further refinements—particularly in chelator conversion efficiency and batch-to-batch consistency—could enhance its translational potential.

In 2024, Garcia-Vallejo et al. [41] reported a novel class of peptide-based MCPs called HyperMAPs (**P-4**), which represent a significant departure from conventional RAFT-polymerized MCPs. By employing SPPS, HyperMAPs achieve monodispersity and precise length control (e.g., 40 repeating units of lysine and 6-aminohexanoic acid). The polymer architecture features three modular components: (1) a metal-chelating backbone (pre-loaded with 21 different metals, including Ln elements and unconventional isotopes like ^93^Nb (niobium) and ^180^Hf (hafnium), using DOTA or DFO chelators), (2) a fluorogenic tracer AMC for conjugation monitoring, and (3) an azide-based click-chemistry handle for antibody linkage. This design enables the incorporation of specific metal isotope ratios, endowing each polymer with a unique CyTOF spectral fingerprint that permits signal deconvolution analogous to spectral flow cytometry. This innovative design enables the precise incorporation of specific metal isotope ratios, endowing each polymer with a unique CyTOF spectral fingerprint that permits signal deconvolution analogous to spectral flow cytometry. In a 13-parameter CyTOF panel, HyperMAPs demonstrated a performance comparable to that of commercial Maxpar polymers while offering superior flexibility in metal loading and reduced NSB. The study underscores the critical importance of scaffold precision in diagnostic probe design, though long-term stability requires further validation. By decoupling metal chelation (through pre-complexation) from polymer assembly, HyperMAPs overcome the limitations of polydispersity and stochastic metal loading inherent to conventional approaches, thereby establishing a versatile platform for expanding multiplexed cytometry panels.

While Ln-chelating polymers have become the mainstay in mass cytometry applications due to their well-defined coordination chemistry and commercial accessibility, their limited channel availability has constrained further development. To enhance multiplexing capabilities and explore new detection channels, researchers are actively developing alternative MCPs incorporating transition metals such as yttrium (Y), zirconium (Zr), rhenium (Re), and Pt. Unlike Ln elements that primarily utilize polyaminocarboxylate-based chelators (e.g., DOTA and DTPA), these transition metals often require customized coordination environments, presenting unique challenges in polymer design.

Zirconium offers four stable isotopes suitable for mass cytometry, and the development of Zr-based reagents holds promise for expanding the detection channels in this technique. The polymer **P-7** features a poly(methacrylamide) backbone strategically functionalized with DFO pendant groups for high-affinity Zr chelation [50]. To address the inherent hydrophobicity of DFO, polyethylene glycol (PEG) side chains were incorporated to enhance aqueous solubility. The design further integrates fluorescein moieties for fluorescence tracking and a terminal biotin group for conjugation to streptavidin-coated microbeads. Despite challenges, including limited polymer length and a modest Zr payload (averaging ~4 ions per chain), the probe generated detectable Zr signals via IMC, confirming the viability of Zr for mass cytometry applications. This work underscores the critical balance between chelator density (hydrophobic DFO) and solubility (hydrophilic PEG) while highlighting the need for longer, more soluble polymers to achieve practical utility in multiplexed assays.

To target soft transition metals like Re and Pt, Winnik’s group developed polyacrylamide-based polymers featuring tailored DPA chelators for effective metal ion coordination [51]. The polymer design incorporated PEG chains at both the backbone terminus and selected chelator-modified side chains to address solubility challenges posed by hydrophobic metal complexes. For Re (**P-8**), a bismaleimide group was introduced at the polymer terminus to enable thiol–maleimide conjugation. By contrast, the platinum-tagged polymer (**P-9**) incorporated azide groups on specific chelator-bearing side chains for DBCO-azide click chemistry, thereby avoiding potential Pt–thiol interactions. Antibody-polymer conjugates of CD20 and CD8a prepared through these coupling strategies demonstrated a performance comparable to commercial Maxpar^®^ reagents in multiplex immunoassays. However, both polymers exhibited some degree of nonspecific cell binding. Additionally, the Pt-Cl bond showed susceptibility to ligand exchange reactions, highlighting a key stability challenge for Pt-based tags.

The structure of Pt-based MCP (**P-9**) was systematically optimized to minimize NSB, as demonstrated in modified structures **P-10** and **P-11** [52]. Key strategies included varying the length of PEG chains attached to DPA (from mPEG_6_ to mPEG_12_ and mPEG_24_) and testing zwitterionic substituents (ZW-NH_2_ and poly(sulfobetaine methacrylate), PSBMA). Notably, mPEG_24_ and PSBMA_29_ significantly reduced NSB, with PSBMA_29_ performing comparably to mPEG_24_. By contrast, the small-molecule zwitterion ZW-NH_2_ increased NSB when attached to DPA. The most effective approach involved ligand exchange with glutathione (GSH), which replaced the Pt-Cl bond with a Pt-S bond, neutralizing the cationic charge and further suppressing NSB. This modification could be combined with PEG or PSBMA_29_ grafting to achieve even greater NSB reduction. Building on these findings, the team synthesized DPA-chelated polyacrylamide polymers (**P-11**, DP = 22 and 55) incorporating mPEG_24_ for hydrophilic shielding, GSH for charge neutralization, and azido-PEG_23_ spacers for efficient antibody conjugation via strain-promoted click chemistry [53]. The polymers demonstrated excellent performance in an 11-plex SMC assay using three platinum isotopes (^195^Pt, ^196^Pt, and ^198^Pt) for peripheral blood mononuclear cell analysis. When applied in a five-plex IMC assay on healthy human tonsil tissue, the ^196^Pt-polymer generated high-resolution images comparable to commercial lanthanide-based reagents. This work highlights the critical importance of balancing polymer length (DP), hydrophilic shielding (mPEG_24_), and charge neutralization (GSH) in probe design while providing a versatile platform to expand mass cytometry multiplexing capabilities and introducing three new mass channels.

### 2.3. Main-Group Metal-Chelating Polymers

Although transition metals (particularly Ln elements) predominantly serve as the foundation of mass cytometry reagents due to their broad mass range and minimal biological interference, certain main-group metals (e.g., In^3+^, and Bi^3+^) have gained attention for their distinctive properties. Polymers functionalized with main-group metal-chelating ligands expand the available detection channels and provide cost-effective alternatives for certain targets. This section explores recent advances in main-group metal-chelating polymer design, their unique challenges, and their role in multiplexed biomarker detection.

Bismuth (Bi) possesses a naturally monoisotopic form (^209^Bi, 100% abundance) with an ionic radius-to-charge ratio nearly identical to lanthanides, enabling Bi^3+^ cations to coordinate with DTPA chelators in the same O/N-donor geometry as lanthanides. Nolan’s group [54,55] developed a robust polymer-based probe design, leveraging the coordination chemistry of Bi^3+^ with DTPA chelators embedded in Maxpar^®^ X8 polymers, which mimics the stable O/N-donor geometry of lanthanide complexes. Furthermore, this polymer platform also demonstrates the effective chelation of Y^3+^. However, the chelation conditions differ significantly: while Ln^3+^ remains stable in pH 5.5–6.0 ammonium acetate buffer, Bi^3+^ requires acidic conditions (2.0–5.0% HNO_3_) to prevent hydrolysis. Post chelation, both Ln@DTPA and Bi@DTPA complexes exhibit stability in neutral or slightly alkaline buffers (e.g., PBS, ideal for maleimide-thiol coupling). Compared to Ln tags, ^209^Bi demonstrates comparable sensitivity (e.g., to ^170^Er) with minimal spectral interference, unlocking the previously unused *m*/*z* 209 channel. This probe’s utility was validated in Human natural killer (NK) cell phenotyping and cell-cycle analysis, showcasing its precision in high-dimensional assays. Future directions may explore hybrid Bi^3+^-incorporating probes to further maximize channel utilization in mass cytometry.

In 2020, Tripier and colleagues [56] successfully enabled the indium (In) channel for mass cytometry by developing an In(III)-chelating polylysine polymer through ROP. The polymer was functionalized with the remarkably stable [In(cb-te2pa)]^+^ complex [57], though with a loading of only 3–4 In chelates per polymer chain. Key structural features included a biotin terminus for efficient conjugation to biotinylated beads or antibodies via biotin/neutravidin interaction, along with a PEG spacer to enhance aqueous solubility. Initial validation studies demonstrated the effectiveness of the probe through high-quality IMC based on ^115^In detection. When conjugated to anti-CD20 antibodies, the probe enabled the specific detection of CD20^+^ Ramos cells in CyTOF assays with excellent signal-to-noise ratios. Notably, the conjugate maintained exceptional stability over three months, highlighting its potential for multiplexed assays. This work exemplifies the strategic combination of kinetically inert metal chelators with modular conjugation approaches to expand the available isotope repertoire in mass cytometry. Future refinements could focus on increasing chelate density to boost signal intensity for detecting lower-abundance biomarkers while maintaining the probe’s favorable stability profile.

## 3. Polymeric Particles as Mass Cytometry Regents

Building on the foundation of MCPs discussed previously, this chapter highlights polymeric particles as alternative and complementary mass tag reagents for cytometry applications. Polymeric particles offer distinct advantages through their particulate nature and structural versatility, including enhanced multiplexing capacity, superior signal amplification, and dual-mode detection capabilities. We focus on two key categories of polymeric particle tags: polymer microspheres (micron-scale and nanoscale) that enable high-throughput multiplexed assays through size-encoded or bio-orthogonal coding strategies, and polymer dots (Pdots, nanoscale) that combine high-density metal loading with intrinsic optical properties for correlative mass/fluorescence cytometry applications. The following sections examine their design principles, synthesis strategies, and applications in single-cell mass cytometry analysis.

### 3.1. Polymer Microspheres

Polymer microsphere-based mass cytometry reagents are functionalized by conjugating metal isotopes (predominantly Ln elements) onto micron-sized beads or NPs. These metal-encoded microspheres serve as multiplexed detection probes in mass cytometry, enabling high-dimensional single-cell analysis. These functional micron-scale microspheres are primarily fabricated through precisely controlled multi-stage dispersion polymerization processes that enable simultaneous control over particle uniformity, metal encoding efficiency, and surface functionality. Two material systems have emerged: lanthanide-encoded PS microspheres synthesized via conventional dispersion polymerization, and poly(methyl methacrylate) (PMMA) microspheres incorporating lanthanide fluoride NPs (LnF_3_ NPs) through photoinitiated RAFT dispersion polymerization. In this section, we detail these synthetic approaches, their respective advantages and limitations, and their applications.

**Figure 9:**
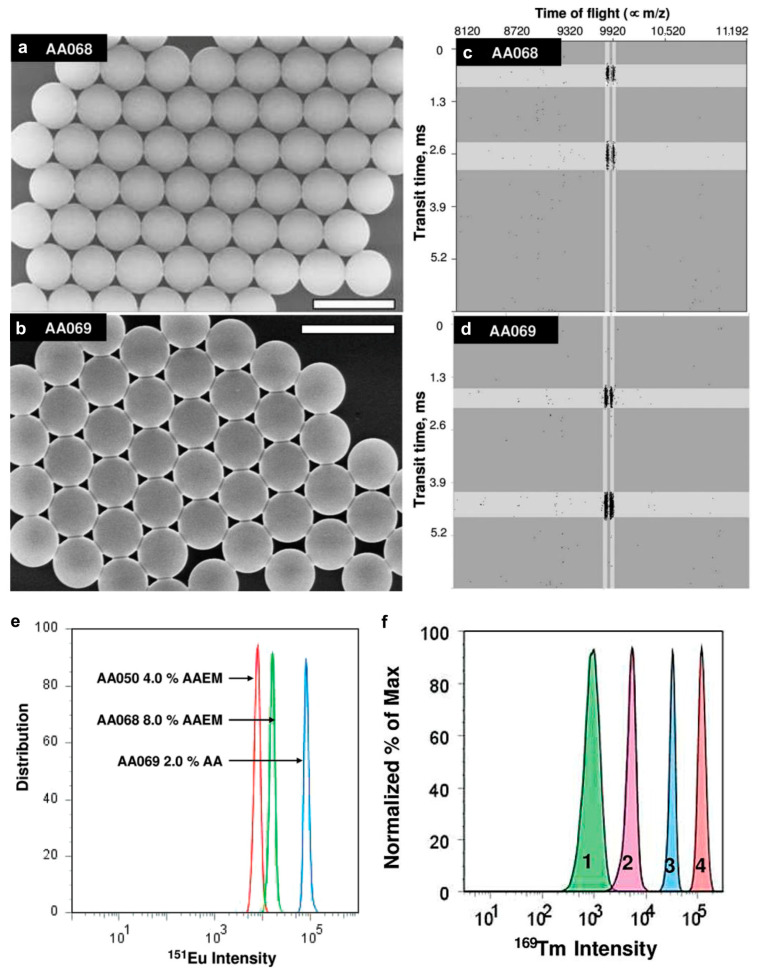
SEM images and mass cytometry screen captures for PS microsphere samples AA068 and AA069 synthesized in the presence of 1% EuCl3 added in the second stage with (**a**,**c**) AAEM: 8 wt%/styrene (*d* = 2.7 μm, *CV*_d_ = 1.5%), and (**b**,**d**) AA: 2 wt%/styrene (*d* = 2.7 μm, *CV*_d_ = 1.4%). The white horizontal bars in the microphotographs represent 5 μm. (**e**) Distribution of mass cytometry signal intensity for three different populations of PS microspheres prepared in presence of EuCl3 (1.0 wt%/styrene) plus (AA050) AAEM (4.0 wt%/styrene, ^151^Eu Intensity = 6900), (AA068) AAEM (8.0% wt%/styrene, ^151^Eu Intensity = 15,100) and (AA069) AA (2.0 wt%/styrene, ^151^Eu Intensity = 90,600). (**f**) Mass cytometry distribution of signal intensity for encoding elements for the bead samples. Beads were encoded with Tm and five levels of concentration (coded from “0” to “4”). Reproduced with permission [58]. Copyright 2009, American Chemical Society.

Metal-encoded micron-scale PS microspheres are synthesized through a multi-stage dispersion polymerization process [58]. The synthesis begins with styrene polymerization in ethanol, followed by the addition of Ln salts (e.g., LnCl_3_) and chelating comonomers (acrylic acid, AA, or acetoacetylethyl methacrylate, AAEM) at approximately 10% monomer conversion (after the particle nucleation stage is complete). The carboxyl groups of AA or β-ketoester groups of AAEM effectively chelate metal ions, yielding uniformly sized microspheres (~2 μm in diameter) with an exceptionally narrow size distribution (*CV*_d_ < 3%) (Figure 9). Notably, AA demonstrates a superior metal-binding capability compared to AAEM, enabling higher Ln loading (10^6^–10^8^ Ln^3+^ ions per bead). These polymer microspheres, prepared via two-stage dispersion polymerization (2-DisP), contain sufficient surface carboxyl groups for antibody conjugation, comparable to commercial beads used in bioassays. However, challenges were encountered in attaching certain biomolecules to 2-DisP-derived particles. To address this limitation, a third reaction stage was introduced at ~60% conversion, incorporating additional AA and a small amount of crosslinker ethylene glycol dimethacrylate. This modification significantly increased the surface carboxyl group density while simultaneously improving the variation in Ln content per particle. The unique encoding capability of metal-encoded microspheres, achieved through the precise control of multiple lanthanide elements at varying concentrations, enables highly multiplexed applications such as antibody-coupled immunoassays, where the simultaneous detection of numerous biomarkers is required.

Ln-encoded poly(styrene-co-methacrylic acid) (P(S-MAA)) microspheres with a uniform diameter of approximately 2 μm and a narrow size distribution were also successfully synthesized through 2-DisP using methacrylic acid (MAA) as a comonomer [59]. The polymerization process demonstrated excellent controllability when incorporating 2% or 4% MAA, yielding monodisperse particles. Under optimal conditions with limited LnCl_3_ salt concentrations, the system achieved a remarkable Ln ion incorporation efficiency exceeding 95% in the presence of 2 wt% MAA. Compared to AA-based systems, the P(S-MAA) microspheres exhibited a significantly higher surface carboxyl group density (up to 38 -COOH/nm^2^), which is crucial for biomolecule conjugation, along with enhanced stability in aqueous buffers. Long-term stability tests demonstrated exceptional performance in neutral buffers (2-(N-morpholino)ethanesulfonic acid, phosphate-buffered saline solution, and ammonium acetate), with minimal Ln ion release (<0.5%) observed over an eight-week period [60]. However, exposure to strong chelating agents (EDTA or DTPA) induced the active extraction of Ln ions, though the total loss remained constrained to less than 15%, indicating that these chelators disrupt the ion–carboxylate interactions within the polymer matrix. The findings provide valuable insights for designing multiplexed detection platforms with minimal signal interference from ion leakage.

**Figure 10:**
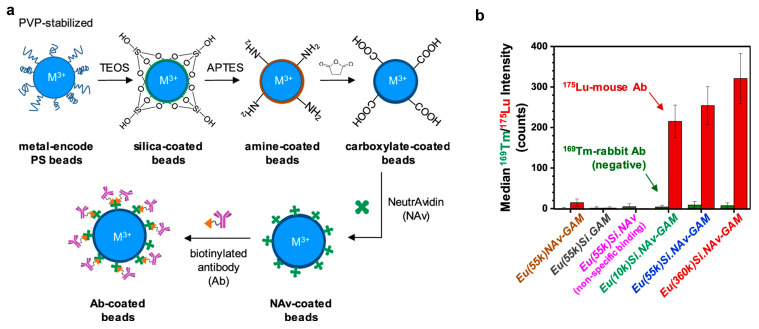
(**a**) Scheme of functionalizing PVP-stabilized metal-encoded microbeads through silica coating. (**b**) Summary of the MC results for detection of Ab reporters using GAM-modified microbeads. Reproduced with permission [62]. Copyright 2021, American Chemical Society.

The polymerizable metal complexes of DTPA derivatives M(DTPA-VBAm_2_) were loaded onto PS beads through a 2-DisP reaction in the presence of polyvinylpyrrolidone (PVP) [61]. This approach enables precise control over the metal content (e.g., Eu) within the microspheres, yielding PS beads with a diameter of approximately 2 μm and a narrow size distribution while achieving a metal complex incorporation efficiency of 60–70%. Proof-of-concept experiments demonstrated that Eu-labeled beads functionalized with goat anti-mouse antibodies effectively detected Lu-tagged mouse IgG. However, surface functionalization with bioaffinity agents remained challenging, likely due to interference from the PVP corona. To address this limitation, a silica coating strategy was introduced, involving the deposition of thin silica shells with tunable roughness and thickness onto the microspheres [62]. As shown in Figure 10, the silica surface was first modified with amine groups, converted to carboxyl groups, and further functionalized with NeutrAvidin to facilitate efficient biotinylated antibody conjugation. The results revealed that microspheres stabilized with higher-molecular-weight PVP exhibited rougher silica surfaces and thicker shells, leading to an enhanced bioconjugation capacity and stronger mass cytometry signals. However, excessive silica thickness introduced noise in CyTOF measurements, underscoring the need for optimized shell thickness.

These polystyrene microspheres loaded with M(DTPA-VBAm2) complexes were further developed into a panel of 11 binary-metal-encoded 3 μm microspheres [63]. These incorporated six lanthanides (La, Ce, Pr, Tb, Ho, Tm) with a precisely controlled metal content, generating consistent median signal intensities of approximately 1000 counts per bead in mass cytometry. Functionalized with target-specific antibodies on their surface, these microspheres effectively captured cytokines in solution, while streptavidin-conjugated gold NPs (AuNPs, 10 nm) served as reporters to identify target cytokine binding. The platform demonstrated robust performance in both four-plex and nine-plex assays, detecting cytokines with picogram-per-milliliter sensitivity in standard solutions and stimulated PBMC samples. However, limitations emerged due to nonspecific interactions, particularly with anti-CD163/CXCL-9 antibodies, necessitating concentration optimization to mitigate interference.

Mei et al. [64] reported a non-Ln metal labeling method for PS microspheres. Their methodology involves incubating commercial polystyrene beads with osmium tetroxide (OsO_4_) to generate OsO_4_-functionalized microspheres. The OsO_4_ covalently binds to unsaturated bonds in the polymer matrix, creating a stable and readily detectable Os isotopic signature while preserving the microspheres’ functional integrity. The Os-labeled microspheres exhibited excellent compatibility with mass cytometry, as their detection did not interfere with other metals commonly used in analytical reagents or sample barcoding, such as Ln, Pd, and Pt. Importantly, the OsO_4_ labeling preserved the antibody-capturing capability of beads, enabling important applications in mass cytometry, including spillover compensation and absolute receptor quantification (e.g., CD4 antibody-binding capacity assays). When combined with the high-dimensional profiling of human PBMCs, these functionalized microspheres allowed for the systematic assessment of receptor expression across diverse cellular phenotypes. This study successfully transformed conventional flow cytometry beads into Os-labeled counterparts compatible with mass cytometry detection, expanding the potential applications of bead-based assays in mass cytometric analyses. Nevertheless, the necessity for rigorous safety measures when handling the hazardous OsO_4_ reagent presents an ongoing challenge for widespread laboratory adoption.

In addition to Ln-encoded PS microspheres, Winnik and colleagues [65] developed functional PMMA microspheres embedded with Ln fluoride NPs (LnF_3_ NPs) for mass cytometry applications. The synthesis employed a photoinitiated RAFT dispersion polymerization strategy in ethanol/water medium, where a carboxy-functional macro-RAFT agent [poly(oligo(ethylene glycol) methyl ether acrylate-co-acrylic acid)trithiocarbonate (P(OEGA-co-AA)-TTC)] served as the stabilizer. The two-stage polymerization process first generated uniform PMMA seed microspheres under UV irradiation, followed by the introduction of LnF_3_ NPs (e.g., LaF_3_, and CeF_3_) along with an additional monomer, enabling nanoparticle incorporation into the microsphere matrix through copolymerization. The resulting microspheres exhibited a high Ln payload (10^5^–10^6^ ions/bead), and surface carboxyl groups for EDC/NHS-mediated bioconjugation (e.g., streptavidin attachment), while the OEGA component effectively minimized nonspecific protein adsorption. This innovative stage-separated approach successfully addressed the challenge of nanoparticle-induced size polydispersity. However, the requirement for methacrylate-functionalized LnF_3_ NPs may limit versatility, and the multi-step synthesis could pose scalability challenges. Future optimizations could explore alternative nanoparticle surface modifications or polymerization techniques to broaden the system’s applicability.

Beyond the aforementioned micron-scale polymer microspheres, polymeric nanospheres have also been developed as mass cytometry reagents. These nanospheres offer distinct advantages due to their smaller size, which facilitates cellular internalization while maintaining relatively large metal-loading capacity. In 2007, Winnik et al. [66] synthesized Ln-containing PS NPs (~100 nm diameter) through miniemulsion polymerization of styrene in the presence of Ln^3+^ complexed with tris-4,4,4-trifluoro-1-(2-naphthyl-1,3-butanedione) ligand. These NPs achieved the loading of approximately 10^3^ Ln^3+^ per particle. The study demonstrated successful nonspecific endocytosis in three leukemia-associated cell lines and enabled quantitative monitoring of phorbol-ester-induced cellular adhesion in suspended THP-1 cells using ICP-MS, establishing polymeric nanospheres as versatile mass tag reagents.

Sanchez-Martin and colleagues [67] developed two stable hybrid-tag nanotrackers by first PEGylating amino-functionalized cross-linked PS NPs, then conjugating them with cyanine fluorophores (Cy3 or Cy5). Isotopically enriched palladium (^106^Pd and ^110^Pd) was subsequently coordinated to the electron-rich network formed between the cyanine polymethine chain and polystyrene aromatic rings, followed by reduction to Pd(0). These polymer NPs exhibit excellent monodispersity, low cytotoxicity, and dual-modal fluorescence/mass detection capabilities, enabling robust live-cell tracking for up to 14 days. The design capitalizes on the tunability and biocompatibility of PS NPs, demonstrating their versatility as universal barcoding reagents independent of cellular protein expression. Importantly, they remain compatible with standardized cytometry reagents and cell processing protocols for mass cytometry. Comparative studies in drug response assays and heterogeneous cell populations reveal significant advantages over conventional barcoding methods. The integration of fluorescence and mass cytometry readouts highlights the innovative synthesis and functionalization strategies employed, significantly expanding the biomedical applications of these NPs.

In 2023, Bai and co-workers [68] also developed a universal mass tag based on PS NPs for mass cytometry. Their approach utilized commercially available carboxyl-functionalized PS NPs (200 nm) loaded with metals (e.g., Ln, Zr, Hf) via a swelling method, where metal acetylacetonates were incorporated into the NPs during solvent evaporation. Antibody conjugation was achieved through EDC/sulfo-NHS chemistry, enabling precise biomarker targeting. An important innovation was the significant reduction in NSB by replacing conventional staining buffers with 10% fetal bovine serum in PBS, eliminating the need for complex surface modifications. The PS NP mass tags demonstrated remarkable sensitivity, outperforming commercial MCPs by five-fold while maintaining compatibility with standard MCP-antibody conjugates for highly multiplexed detection of human mononuclear cell biomarkers. Furthermore, Hf-doped PS-NPs unlocked four new mass cytometry detection channels (^177^Hf, ^178^Hf, ^179^Hf, and ^180^Hf), substantially expanding the multiplexing capacity of the technique.

### 3.2. Polymer Dots

Pdots have emerged as a novel class of mass cytometry probes, combining nanoscale dimensions (20–30 nm) with superior photophysical properties. These nanoscale fluorescent probes combine the advantages of high brightness, photostability, and surface functionality, making them particularly suitable for advanced cytometry applications where both optical and mass detection are required. The development of Ln-coordinated Pdots (Ln-Pdots) represents a significant advancement in this field, enabling dual-modal detection through their unique combination of fluorescent semiconducting polymers and mass-detectable Ln elements.

**Figure 11:**
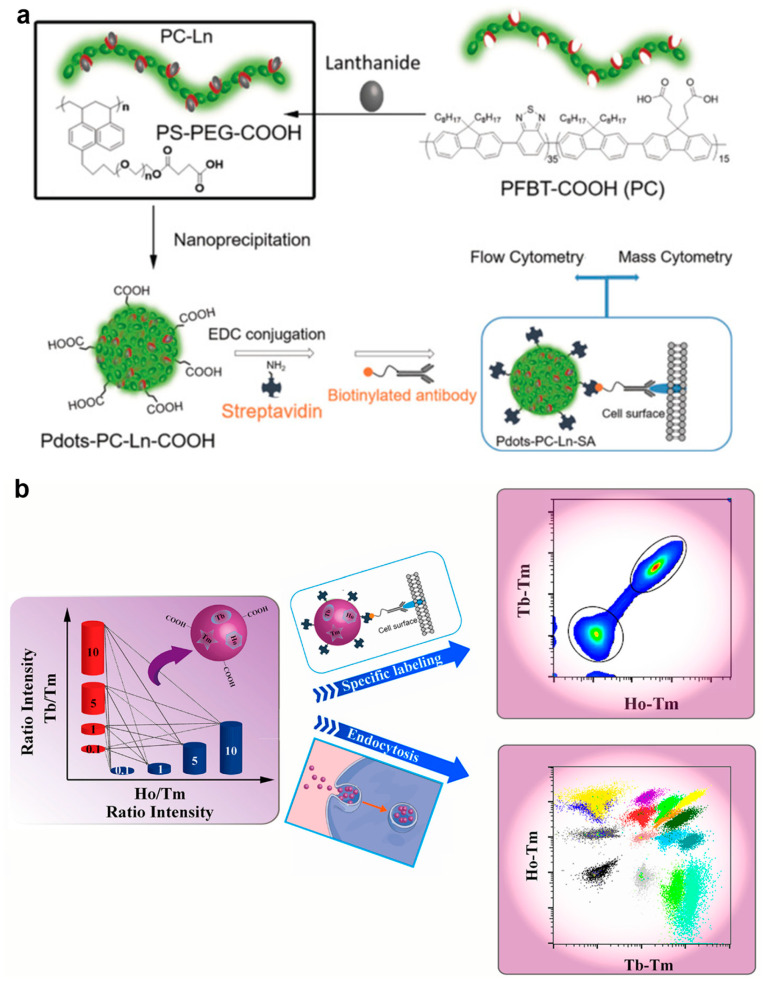
(**a**) Schematic depiction of the preparation, bioconjugation, and cell labeling of dual-functional Pdots for flow cytometry and mass cytometry. Reproduced with permission [69]. Copyright 2017, John Wiley and Sons. (**b**) Schematic showing the ratiometric barcodes based on Ln-Pdots for multiplex mass cytometric analysis. Reproduced with permission [70]. Copyright 2018, American Chemical Society.

In 2017, Chiu’s group [69] developed Ln-coordinated Pdots (Ln-Pdots) as dual-functional probes for flow cytometry and mass cytometry. As shown in Figure 11a, the synthesis begins with the chelation of carboxyl-functionalized poly[(9,9-dioctylfluorenyl-2,7-diyl)-co-(1,4-benzo-{2,10,3}-thiadiazole)] polymer (PC) with lanthanide ions (Nd^3+^, Eu^3+^, Tb^3+^, Ho^3+^, or Er^3+^) in THF for 1 h to form Ln–polymer complexes. This system is then mixed with the block copolymer, polystyrene-grafted ethylene oxide functionalized with carboxyl groups (PS-PEG-COOH), and rapidly injected into water, where nanoprecipitation yields 20–30 nm Ln-Pdots (Pdot-PC-Ln-COOH). Through optimization of the polymer-to-Ln molar ratio (e.g., carboxyl: Eu^3+^ = 2:1) and PS-PEG-COOH content (40 wt%), the resulting Pdots achieve both small size and high Ln loading (approximately 1100–2000 Ln atoms per Pdot). The subsequent conjugation of surface carboxyl groups with streptavidin produces dual-functional probes (Pdot-PC-Ln-SA) suitable for cell labeling. This approach maintains the excellent fluorescent properties of Pdots (with quantum yields up to 19%) while significantly enhancing mass cytometry signals through high-density Ln labeling. The experimental results demonstrate superior performance in both flow cytometry and mass cytometry applications. When tested with MCF-7 breast cancer cells and PBMC models, the Pdot probes not only delivered strong fluorescence signals for flow cytometry but also outperformed commercial reagents in mass signal intensity while maintaining low NSB. However, the relatively broad size distribution of current Pdots may affect batch-to-batch consistency, representing an area for future optimization. This work establishes an important platform for developing advanced probes that bridge optical and mass cytometry techniques.

Building on this work, the team subsequently developed a ratiometric barcoding strategy based on Ln-Pdots for mass cytometry [70]. The barcoded Ln-Pdots were prepared following a protocol similar to previous syntheses but with modifications; specifically, PC was coordinated with a mixture of three lanthanide ions (Tb, Ho, Tm) at different ratios. As illustrated in Figure 11b, this innovative approach generated 16 distinct barcodes by employing three lanthanide isotopes and four intensity ratios (0.1, 1, 5, 10), significantly enhancing multiplex sample analysis efficiency. Unlike conventional absolute-intensity-based barcoding, this ratiometric design used Tm as an internal reference (with Tb/Tm and Ho/Tm ratios as encoding parameters), effectively eliminating biases caused by variations in labeling efficiency, reagent concentration, or instrument fluctuations, thereby improving data reliability. This system successfully distinguished the 16 sets of labeled MCF-7 cells with mass cytometry through endocytic uptake and demonstrated practical utility in PBMC studies via CD45-specific labeling. While maintaining the advantages of low cytotoxicity and dual-labeling capacity from the original design, the Ln-Pdots still face challenges in size uniformity and require a Cell-ID intercalator for cell doublet discrimination.

## 4. Metal–Organic Framework as Mass Cytometry Regents

MOFs have recently emerged as a promising class of mass tags for high-dimensional cytometry, demonstrating significant advantages in both metal-loading capacity and structural tunability [71,72]. These crystalline porous materials, formed through the self-assembly of metal ions with organic linkers, provide an optimal scaffold for incorporating metal isotopes at high densities, thereby significantly enhancing mass cytometry detection sensitivity. Their inherent modularity enables precise control over composition, porosity, and surface functionality, allowing for tailored designs to meet specific biological application requirements [73,74,75]. This section reviews recent advances in MOF-based mass tags, with particular emphasis on their unique structural characteristics, superior performance in multiplexed cellular analysis, and emerging strategies to optimize their biocompatibility and labeling efficiency for single-cell mass spectrometry. The development of MOF mass tags represents a paradigm shift in cytometry reagent design, overcoming numerous limitations of conventional polymer-based tags while creating new possibilities for high-parameter single-cell analysis.

**Figure 12:**
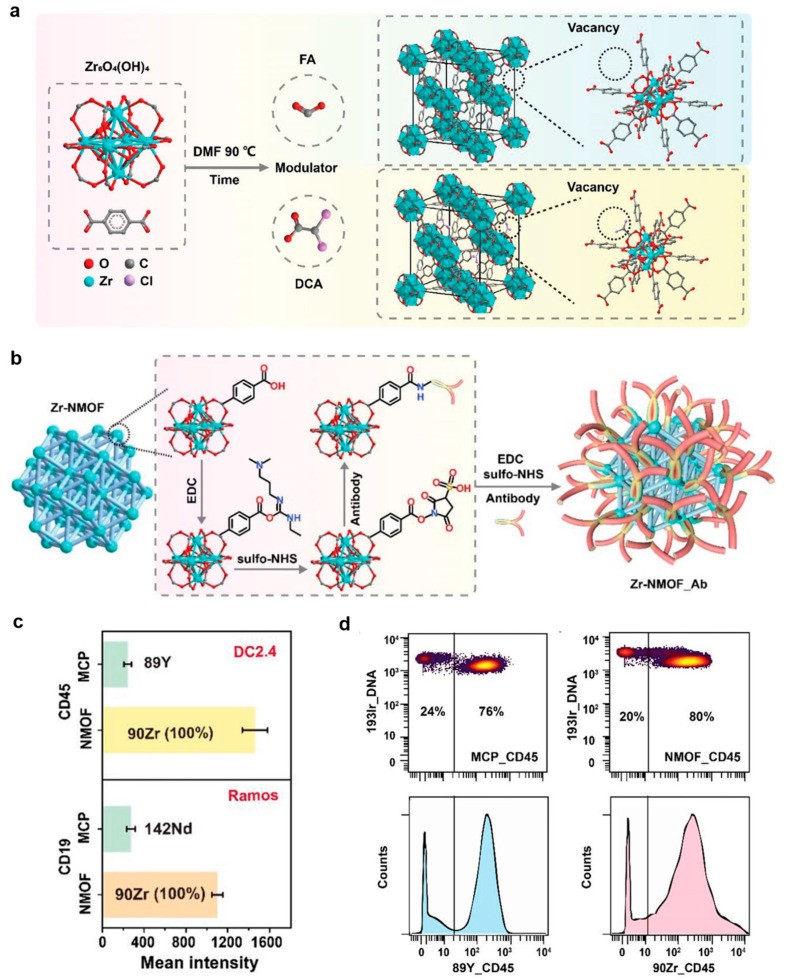
Schematic illustration for (**a**) carboxylic acid modulated synthesis of Zr-NMOF, and (**b**) Zr-NMOF functionalization with antibody via EDC/sulfo-NHS reaction. (**c**) Sensitivity of Zr-NMOF_Abs compared to MCP-Abs. Stain panel: 89Y-MCP-CD45, Zr-NMOF_CD45 (3000 NPs/cell) for DC2.4 staining; 142Nd-MCP-CD19, Zr-NMOF_CD19 (3000 NPs/cell) for Ramos staining. (**d**) Contour plots and corresponding histogram of cell mixture (B16:DC2.4 = 1:3) stained by MCP-89Y_CD45 and Zr-NMOF_CD45(1500 NPs/cell). Reproduced with permission [76]. Copyright 2021, John Wiley and Sons.

In 2021, Ding et al. [76] pioneered the use of zirconium-based nanoscale metal–organic frameworks (Zr-NMOFs) as novel mass tags for mass cytometry. As illustrated in Figure 12a, the Zr-NMOFs were synthesized via a solvothermal method using Zr clusters (Zr_6_O_4_(OH)_4_) and terephthalic acid (H_2_BDC) as building blocks, with monocarboxylic acid modulators (formic acid or dichloroacetic acid) to precisely control nucleation and crystal growth. Through the systematic optimization of modulator concentration (e.g., 0.9 M formic acid) and reduction in reaction time to 6 h, the team successfully obtained a uniform-sized Zr-NMOF (33 nm) with narrow size distribution (PDI < 0.1) and exceptionally high metal-loading capacity (~10^5^ Zr ions per particle). The resulting Zr-NMOFs demonstrated remarkable colloidal stability in aqueous solutions for over one year and maintained structural integrity during antibody conjugation via EDC/sulfo-NHS chemistry (Figure 12b). The design capitalized on the porous crystalline structure of UIO-66 and functionalized surface chemistry, incorporating four stable Zr isotopes (*m*/*z* = 90, 91, 92, 94) to expand detectable mass channels. In multiparameter assays for mouse spleen cell staining, the Zr-NMOF tags showed excellent compatibility with conventional MCP tags while delivering a five-fold greater signal amplification (Figure 12c). Furthermore, in cell mixture experiments with DC2.4 and B16 cells at a 3:1 ratio, the antibody-functionalized Zr mass tags exhibited a cell-targeting specificity comparable to that of standard MCP tags (Figure 12d). This groundbreaking work established MOFs as powerful mass tags, overcoming the limitations of MCPs in metal density and channel availability, while opening new possibilities for high-dimensional immune profiling and advanced barcoding applications.

**Figure 13:**
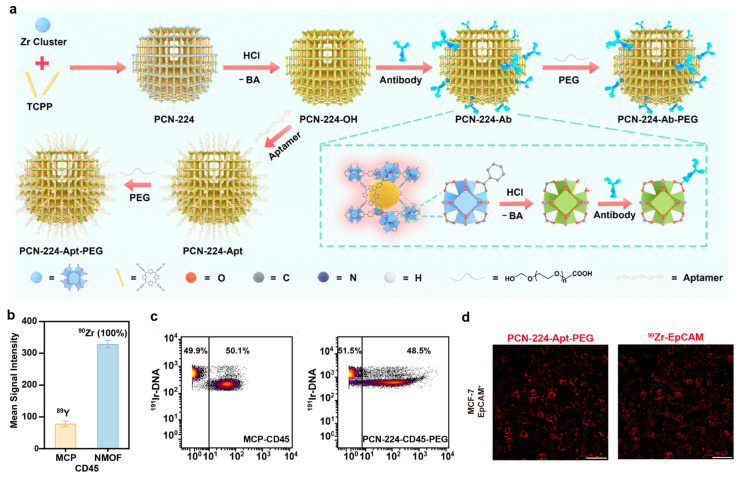
(**a**) Synthesis of metallic and fluorescent dual-property PCN-224-Ab-PEG and PCN-224-Apt-PEG probes. (**b**) Sensitivity of PCN-224-CD45-PEG (40k NPs/cell) compared to that of 89Y-MCP-CD45. (**c**) Contour plots of cell mixture (DC 2.4/Ramos = 1:1) stained by 89Y-MCP-CD45 and PCN-224-CD45-PEG (40k NPs/cell). (**d**) IFM and IMC images of MCF-7 stained by PCN-224-Apt-PEG. Reproduced with permission [77]. Copyright 2025, American Chemical Society.

Building upon this foundation, this team recently developed a class of dual-functional probes based on luminescent PCN-224-OH material [77]. They synthesized the light-emitting MOF (PCN-224-OH) using tetrakis(4-carboxyphenyl)porphyrin (TCPP) as the organic ligand and Zr_6_O_4_(OH)_4_ as metal nodes, replacing H_2_BDC (Figure 13a). Subsequent hydrochloric acid (HCl) treatment effectively removed surface-bound benzoate groups while preserving the structural integrity of MOF, exposing reactive Zr-OH^−^/H_2_O sites. This PCN-224-OH with abundant surface coordination sites enables direct antibody/aptamer conjugation through carboxyl or phosphate groups, eliminating the need for additional coupling chemistry. To enhance dispersion stability and minimize nonspecific protein interactions, PCN-224-Ab/Apt was further modified with PEG, resulting in PCN-224-Ab/Apt-PEG probes. This innovative design integrates Zr clusters with fluorescent TCPP ligands, allowing for simultaneous fluorescence and mass signal detection without complex post-synthetic modifications. The porphyrin-based framework prevents aggregation-caused quenching (ACQ) while ensuring excellent photostability. In practical applications, the PCN-224-Ab-PEG probe demonstrated a 4.2-fold higher sensitivity than that of conventional MCP (89Y-MCP-CD45) in CyTOF while maintaining exceptional specificity (Figure 13b,c). Meanwhile, the PCN-224-Apt-PEG probe enabled rapid region-of-interest (ROI) localization through fluorescence imaging, followed by high-parameter mass cytometry imaging at targeted ROIs (Figure 13d), reducing the IMC scanning time by 90%. This dual-modal approach significantly enhances both the efficiency and precision of single-cell analysis.

**Figure 14:**
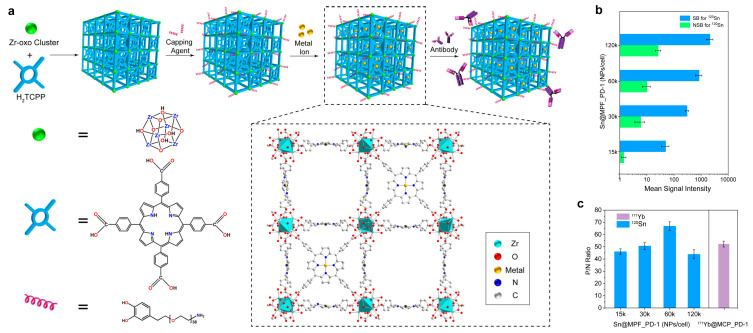
(**a**) Schematic illustration for the structure and the synthesis procedure of the MPF-based CyTOF markers. (**b**) MSI of 120Sn for staining Ramos cells and Jurkat cells with Sn@MPF_PD-1 at different staining ratios (15k to 120k NPs/cell). The error bar represents one standard deviation, *n* = 3. (**c**) MSI^+^ to MSI^−^ (P/N) ratio for the concentration titration of mixed cells with a Ramos-to-Jurkat ratio of 50:50. The error bar represents one standard deviation, *n* = 3. Reproduced with permission [78]. Copyright 2022, John Wiley and Sons.

The application of TCPP in constructing Zr-MOF-based mass cytometry reagents was pioneered by Luo et al. [78], who developed functionalized mesoporous porphyrinic frameworks (MPFs) as scaffolds for chelating non-lanthanide metals (Sn, Pt, W, Pd). As illustrated in Figure 14, the synthesis of MPF-based CyTOF markers begins with the preparation of PCN-224 nanoparticles (<40 nm) by dissolving ZrOCl_2_·8H_2_O and benzoic acid in DMF, followed by the addition of pre-dissolved TCPP ligands. To enhance colloidal stability and provide reactive amine groups for subsequent functionalization, the PCN-224 NPs were then surface-modified and capped with 4-(amino-polyethylene glycol-ethyl)benzene-1,2-diol (APEB). This critical PEGylation step prevents nanoparticle aggregation during subsequent processes, including metal loading into the porphyrin centers and antibody conjugation via *N*-succinimidyl 4-(*N*-maleimidomethyl) cyclohexanecarboxylate coupling. The porphyrin centers enabled relatively high-density metal loading, achieving superior sensitivity compared to commercial polymer-based markers while maintaining low NSB. Furthermore, these MPF-based markers demonstrate excellent compatibility with commercial CyTOF tags in co-staining experiments, enabling high-throughput multiparameter analysis of heterogeneous populations in human PBMCs. This innovative approach addresses a key limitation in mass cytometry by rationally engineering probes to access previously underutilized detection channels. Future research directions may explore non-Zr frameworks to further expand the available isotope repertoire for enhanced multiplexing capabilities.

In 2024, Bai et al. [79] developed an innovative mass cytometry barcoding strategy leveraging the intrinsic positive charge of nano-sized UIO-66(Zr/Hf) MOFs for universal cell labeling through nonspecific electrostatic binding. While employing a synthesis method similar to conventional MOF probes (solvothermal reaction with formic acid modulation), this work uniquely capitalized on the strong cationic surface properties of UIO-66 (zeta potential: 32 mV for Zr, 42 mV for Hf) to overcome traditional marker-dependent limitations. The strategy enabled 84 distinct barcode combinations (9-choose-3) by utilizing nine naturally abundant Zr and Hf isotopes, demonstrating a 16-fold higher sensitivity than commercial polymer tags while maintaining full compatibility with standard MCP probes for the multiplexed detection of human leukocyte biomarkers. The versatility of this platform was further enhanced through fluorescent dye conjugation, permitting dual mass/fluorescence detection modalities. By strategically repurposing typically undesirable nonspecific binding effects, this study repositions MOFs as multifunctional barcoding tools that preserve native protein expression profiles during high-throughput analysis.

## 5. Conclusions and Outlook

Mass cytometry has transformed single-cell analysis by employing stable heavy metal isotopes as mass tags, overcoming the spectral limitations inherent to conventional fluorescence-based flow cytometry. Polymer-based reagents, including MCPs, polymeric particles, and MOFs, have played critical roles in this advancement. Each reagent class exhibits unique functional advantages while presenting distinct technical challenges. Below, we provide a critical comparative analysis of their respective merits and limitations.

MCPs represent the most mature technology, with commercial Maxpar^®^ reagents serving as the current gold standard due to their well-defined synthesis and robust performance. Their key advantages include the following: (1) well-established polymerization techniques (e.g., RAFT, ROP, SPPS) that enable control over polymer architecture and metal incorporation; (2) excellent performance in antibody labeling and cell staining applications; and (3) consistent reliability in routine immunophenotyping. However, their applications remain largely confined to trivalent metal isotopes (particularly Ln), primarily due to the selectivity limitations of existing chelating ligands. More notably, the fundamental constraint on the total number of metal ions that can be chelated by the side chains of a single MCP molecule substantially limits further improvements in detection sensitivity. Furthermore, the synthesis of perfectly monodisperse MCPs remains challenging due to the inherent limitations of RAFT and ROP polymerization techniques, which offer only moderate control over polymer chain growth.

Polymeric particles, including polymer microspheres and Pdots, offer several significant advantages for mass cytometry applications: (1) Their metal-loading capacity (10^3^–10^8^ ions/particle) far exceeds that of conventional MCPs, enabling substantial signal amplification. (2) Their dual fluorescence/mass detection capabilities in nanoscale polymer microspheres and Pdots enable simultaneous fluorescence and mass cytometry measurements. (3) Their ability to incorporate multiple Ln isotopes significantly expands multiplexing potential. However, these materials face several limitations: batch-to-batch variability due to inconsistent particle sizes, restricted metal-tag diversity from predominant reliance on Ln isotopes, and an increased risk of nonspecific binding resulting from their high surface area (necessitating surface modification with PEG or other passivation agents). Furthermore, unlike commercially available MCPs, most polymeric particle reagents remain primarily confined to research laboratories, with limited options for standardized commercial procurement.

MOFs represent a promising emerging class of mass cytometry tags, distinguished by several advantages: (1) Their high metal density delivers superior detection sensitivity in mass cytometry. (2) Their modular design allows for tailored control over composition, porosity, and surface functionality to meet specific biological application requirements. (3) Their extended crystalline framework provides opportunities for incorporating non-traditional metal isotopes beyond lanthanides, potentially expanding the available mass channels beyond current limitations. Despite these promising features, current MOF technologies face several hurdles. Most prototype MOF tags are based on zirconium frameworks (e.g., UIO-66), which present significant challenges in terms of scalable production. The reproducibility of synthesis remains problematic due to sensitivity to subtle variations in reaction conditions. Maintaining colloidal stability in complex biological matrices while preserving structural integrity is particularly challenging.

To address these limitations, future development should focus on five important areas: (1) the development of novel polymer architectures, combined with the customization or derivatization of macrocyclic/acyclic chelators, which enables the expansion of the metal isotope repertoire beyond Ln to enhance multiplexing capabilities; (2) optimizing surface chemistry of polymeric particles and MOF-based reagents to minimize NSB and improve stability in biological environments, which is important for clinical translation; (3) developing dual-functional probes that combine mass cytometry with fluorescence imaging or spectral flow cytometry to provide complementary datasets for deeper biological insights; (4) streamlining synthesis protocols and establishing standardization for polymer-based mass cytometry reagents to facilitate widespread adoption in both research and clinical settings; and (5) exploring novel applications beyond immunology, such as in cancer research, infectious diseases, and neuroscience. By addressing the current limitations and leveraging interdisciplinary advancements, polymer-based mass cytometry reagents will continue to push the boundaries of high-dimensional single-cell analysis, serving as powerful tools for transformative discoveries in biology and medicine.

## Figures and Tables

**Table 1:** Table of isotopic natural abundances of metal elements used in MCPs [42].

Element	Isotope	Abundance (%)	Element	Isotope	Abundance (%)
Lanthanum	^138^La	0.09	Ytterbium	^168^Yb	0.13
	^139^La	99.91		^170^Yb	3.04
Cerium	^136^Ce	0.185		^171^Yb	14.28
	^138^Ce	0.251		^172^Yb	21.83
	^140^Ce	88.450		^173^Yb	16.13
	^142^Ce	11.114		^174^Yb	31.83
Praseodymium	^141^Pr	100		^176^Yb	12.76
Neodymium	^142^Nd	27.2	Lutetium	^175^Lu	97.41
	^143^Nd	12.2		^176^Lu	2.59
	^144^Nd	23.8	Yttrium	^89^Y	100
	^145^Nd	8.3	Zirconium	^90^Zr	51.45
	^146^Nd	17.2		^91^Zr	11.22
	^148^Nd	5.7		^92^Zr	17.15
	^150^Nd	5.6		^94^Zr	17.38
Samarium	^144^Sm	3.07		^96^Zr	2.80
	^147^Sm	14.99	Niobium	^93^Nb	100
	^148^Sm	11.24	Palladium	^102^Pd	1.02
	^149^Sm	13.82		^104^Pd	11.14
	^150^Sm	7.38		^105^Pd	22.33
	^152^Sm	26.75		^106^Pd	27.33
	^154^Sm	22.75		^108^Pd	26.46
Europium	^151^Eu	47.81		^110^Pd	11.72
	^153^Eu	52.19	Cadmium	^106^Cd	1.25
Gadolinium	^152^Gd	0.20		^108^Cd	0.89
	^154^Gd	2.18		^110^Cd	12.49
	^155^Gd	14.80		^111^Cd	12.80
	^156^Gd	20.47		^112^Cd	24.13
	^157^Gd	15.65		^113^Cd	12.22
	^158^Gd	24.84		^114^Cd	28.73
	^160^Gd	21.86		^116^Cd	7.49
Terbium	^159^Tb	100	Hafnium	^174^Hf	0.16
Dysprosium	^156^Dy	0.06		^176^Hf	5.26
	^158^Dy	0.10		^177^Hf	18.60
	^160^Dy	2.34		^178^Hf	27.28
	^161^Dy	18.91		^179^Hf	13.62
	^162^Dy	25.51		^180^Hf	35.08
	^163^Dy	24.90	Rhenium	^185^Re	37.40
	^164^Dy	28.18		^187^Re	62.60
Holmium	^165^Ho	100	Platinum	^190^Pt	0.014
Erbium	^162^Er	0.14		^192^Pt	0.782
	^164^Er	1.61		^194^Pt	32.967
	^166^Er	33.61		^195^Pt	33.832
	^167^Er	22.93		^196^Pt	25.242
	^168^Er	26.78		^198^Pt	7.163
	^170^Er	14.93	Indium	^113^In	4.29
Thulium	^169^Tm	100		^115^In	95.71
			Bismuth	^209^Bi	100

## Data Availability

No new data were created or analyzed in this study. Data sharing is not applicable to this article.

## Abbreviations

The following abbreviations are used in this manuscript:

TOF-MS	Time-of-flight mass spectrometry
CyTOF	Time-of-flight cytometry
ICP-TOF-MS	Inductively coupled plasma time-of-flight mass spectrometry
MC	Mass cytometry
SMC	Suspension mass cytometry
IMC	Imaging mass cytometry
MCP	Metal-chelating polymer
*m*/*z*	Mass-to-charge ratio
MOF	Metal–organic framework
RAFT	Reversible addition–fragmentation chain transfer
Ln	Lanthanide
ITC	Isothermal titration calorimetry
DOTA	1,4,7,10-Tetraazacyclododecane-1,4,7,10-tetraacetic acid
DTPA	Diethylenetriaminepentaacetic acid
TEPA	Tetraethylenepentamine
EDTA	Ethylenedia minetetraacetic acid
TTHA	Triethylenetetramine-*N*,*N*,*N*′,*N*′′,*N*′′′,*N*′′′-hexaacetic acid
DPA	2,2′-Dipicolylamine
DFO	Deferoxamine
PtBA	Poly(*t*-butyl acrylate)
TFA	Trifluoroacetic acid
PAA	Poly(acrylic acid)
BOC	*t*-Butyloxy carbonyl
DTT	Dithiothreitol
SPPS	Solid phase peptide synthesis
TCO	Transcyclooctene
SPAAC	Strain-promoted alkyne-azide cycloaddition
DBCO	Dibenzocyclooctyne
MAP	Aptamer nanoprobe
PBMC	Peripheral blood mononuclear cell
NSB	Nonspecific binding
PEG	Polyethylene glycol
PSBMA	Poly(sulfobetaine methacrylate)
GSH	Glutathione
NK	Natural killer
NPs	Nanoparticles
Pdots	Polymer dots
PS	Polystyrene
PMMA	Poly(methyl methacrylate)
AA	Acrylic acid
AAEM	Acetoacetylethyl methacrylate
2-DisP	Two-stage dispersion polymerization
P(S-MAA)	Poly(styrene-*co*-methacrylic acid)
MMA	Methacrylic acid
PVP	Polyvinylpyrrolidone
Ln-Pdots	Ln-coordinated Pdots
PC	Poly[(9,9-dioctylfluorenyl-2,7-diyl)-*co*-(1,4-benzo-{2,10,3}-thiadazole)] polymer
Zr-NMOFs	Zirconium-based nanoscale metal–organic frameworks
H_2_BDC	Terephthalic acid
ACQ	Aggregation-caused quenching
ROI	Region-of-interest
MPF	Mesoporous porphyrinic framework
APEB	4-(Amino-polyethylene glycol-ethyl)benzene-1,2-diol
